# Evaluation of the Radiation-Protective Properties of Bi (Pb)–Sr–Ca–Cu–O Ceramic Prepared at Different Temperatures with Silver Inclusion

**DOI:** 10.3390/ma15031034

**Published:** 2022-01-28

**Authors:** Essia Hannachi, K. A. Mahmoud, Aljawhara H. Almuqrin, M. I. Sayyed, Yassine Slimani

**Affiliations:** 1 Department of Nuclear Medicine Research, Institute for Research and Medical Consultations (IRMC), Imam Abdulrahman Bin Faisal University, Dammam 31441, Saudi Arabia; mohammed.alsyyed@iu.edu.jo; 2 Department of Nuclear Power Plants and Renewable Energy, Ural Federal University, St. Mira 19, 620002 Yekaterinburg, Russia; kmakhmud@urfu.ru; 3 Nuclear Materials Authority, El-Maadi, Cairo P.O. Box 530, Egypt; 4 Department of Physics, College of Science, Princess Nourah Bint Abdulrahman University, Riyadh 11671, Saudi Arabia; ahalmoqren@pnu.edu.sa; 5 Department of Physics, Faculty of Science, Isra University, Amman 11622, Jordan; 6 Department of Biophysics, Institute for Research and Medical Consultations (IRMC), Imam Abdulrahman Bin Faisal University, Dammam 31441, Saudi Arabia; yaslimani@iau.edu.sa

**Keywords:** ceramic, phase formation, silver, Monte Carlo, linear attenuation coefficient, radiation shielding efficiency factor

## Abstract

The influences of the sintering process and AgNO_3_ addition on the phase formation and radiation shielding characteristics of Bi_1.6_Pb_0.4_Sr_2_Ca_2_Cu_3_O_10_ were studied. Three ceramics (code: C0, C1, and C2) were prepared as follows: C0 was obtained after calcination and only one sintering step, C1 was obtained after calcination and two sintering cycles, and C2 was prepared after the addition of AgNO_3_ at the beginning of the final sintering stage. C2 displayed the maximum volume fraction of the Bi-2223 phase (76.4 vol%), the greatest crystallite size, and high density. The linear mass attenuation coefficient (µ) has been simulated using the Monte Carlo simulation. The µ values are high at 15 keV (257.2 cm^−1^ for C0, 417.57 cm^−1^ for C1, and 421.16 cm^−1^ for C2), and these values dropped and became 72.58, 117.83 and 133.19 cm^−1^ at 30 keV. The µ value for the ceramics after sintering is much higher than the ceramic before sintering. In addition, the µ value for C2 is higher than that of C1, suggesting that the AgNO_3_ improves the radiation attenuation performance for the fabricated ceramics. It was demonstrated that the sintering and AgNO_3_ addition have a considerable influence on the ceramic thickness required to attenuate the radiation.

## 1. Introduction

At present, high-temperature superconductors (HTS) are one of the most examined progressive materials, owing to their promising standpoints in a variety of technology and science fields [1]. Numerous applications will be technologically advanced in radioactive surroundings, such as nuclear fusion reactors, spatial investigations, or particle accelerators, etc., where radiation can produce severe alteration in material characteristics. Predicting and controlling these alterations is within the scope of many specialists devoted to studying the influences of radiation on superconducting materials.

For example, HTS systems are utilized as protective materials in fusion reactors (FR) [2]. Therefore, it is very significant to examine the efficiency of neutron and gamma protecting superconducting materials for their promptly accessible radiation properties. Linear attenuation coefficient, the half-value layer, and radiation protecting efficiency are basic quantities required to study interactions. These parameters are reliant on the incident energy and the type and quality of the absorbing substances. Furthermore, photon accumulation factors (which depend on energy, thickness, and chemical compositions) are critical parameters that are necessitated to effectively protect the mixture or compound. In FR, extreme energy (~14 MeV) and high density of neutrons is induced during the tritium–deuterium fusion reaction. High energy neutrons cause diverse reactions with fusion reactor building materials to emit γ rays with energies of up to 10–20 MeV [3]. 

Among the well-known HTS, Bi_2_Sr_2_Ca_n-1_Cu_n+1_O_2n+6_ (BSCCO for brevity; *n* = 1, 2, and 3), which involves bismuth, strontium, calcium, and copper oxide, is an interesting class of high-conductive systems. It is known that this system has three phases, 2201, 2212, and 2223, according to *n* value. Of these phases, phases 2212 (*n* = 1) and 2223 (*n* = 2) are more significant owing to their critical temperatures exceeding the liquid nitrogen boiling point [4]. In addition to their high critical temperature, this system exhibits many other advantages, including high critical current, the nonexistence of dust rare earth elements, water resistance, purity, and abundance of superconducting elements. Many researchers have devoted study to the effects of sintering temperature, sintering duration, doping, and pressure on the formation of highly stable BSCCO materials [5,6,7,8]. Various kinds of chemical and physical characteristics of this type of HTS have been investigated [9,10,11]. Yet, their radiation shielding efficiency is very scarce in the literature, unlike the shielding properties of concretes [12,13], glass, and alloys [14,15,16,17]. In a recent study, Singh et al. [18] studied the radiation shielding performances of some superconducting ceramics, including non-centrosymmetric (NCS), iron-based, and oxide-based superconductors. It was found that an iron-based superconductor is efficient as a fast-neutron shielding material for neutron energies ranging from 2 to 12 MeV, while NCS and oxide-based superconductors are superior gamma-rays shielding materials. 

In the current work, we aim to study the structure development and the radiation shielding traits of (Bi_1.6,_Pb_0.4_) Sr_2_Ca_2_Cu_3_O_10_ ceramic prepared under different sintering cycles and with AgNO_3_ addition during the final sintering step. AgNO_3_ has been widely used as the most suitable dopant to improve the physical properties of almost all superconducting materials. The positive effects of silver addition on the physical properties of ceramic superconductors are to promote crystallization, mechanical toughness, and oxygen diffusion [19,20]. For instance, Abdeen et al. [21] showed that the addition of nano-silver to the superconducting phase improved the volume fraction, superconducting, and mechanical properties of the material. In addition, the addition of silver results in the growth of grains leading to the reduction in grain boundaries and porosity and the improvement of intergranular links [22,23]. For example, D. Sýkorová et al. studied the effect of the addition of different amounts of silver (5, 10, and 15 wt.%) on the performance of Bi-based superconductors, and they demonstrated that 15 wt.% of silver addition is the optimal amount for the formation of a low porous and high dense sample with good superconducting properties [24]. Thus, it is expected that superconducting ceramics with the desired radiation shielding properties will be obtained.

Based on this literature review, the phase formation and radiation shielding characteristics of the different proposed ceramics are compared and discussed. The results obtained can be advantageous in diverse applications of superconductors for radiation protecting applications.

## 2. Experiment

### 2.1. Synthesis Protocol and Experimental Characterization

A bismuth-based ceramic with a nominal composition of Bi_1.6_Pb_0.4_Sr_2_Ca_2_Cu_3_O_10_ was fabricated by the solid-state route. The raw powders are consistent with CaCO_3_ (99.9%), SrCO_3_ (99.9%)_,_ and CuO (99.9%). A stoichiometric mixture of these starting powders was subjected to calcination in the air for 24 h at 930 °C to form an oxide precursor without any remainders of carbonates. Then, the precursor oxide was mixed with PbO and Bi_2_O_3_ powders (0.4 Pb per formula unit) and placed into a boat crucible alumina before being heat-treated in the air in a tubular furnace at 835 °C for 140 h, and finally fast cooled to room temperature to produce an intermediate compound, Bi_1.6_Pb_0.4_Sr_2_Ca_2_Cu_3_O_10_. The resulting intermediate compound (IC; referred to as C0) is dark. During the second heat treatment, 15 wt.% of AgNO_3_ was added to the IC. Both powders were thoroughly mixed and ground by hand using an agate mortar with pestle to form a homogeneous mixture. The 0 wt.% powder was also considered, so it was hand-ground as with the powder added with AgNO_3_ to certify similar surrounds for all the ceramics. This 0 wt.% powder was considered as a reference (referred to as C1) and it was compared with C0 (the one that has not subjected to the second heat treatment stage) and with powder prepared with silver addition (referred to as C2). Both powders were then subjected to a sintering step at 850 °C in the air for 156 h after being uniaxially pressed into pellets at 17 MPa. The pellets were then slowly cooled at a rate of 13 °C/min to form a dense and well-compacted ceramic. Table 1 illustrates the ceramic code with their sintering conditions and their density values.

X-ray powder diffraction (XRD; Philips 1710 diffractometer) was performed using CuKα radiations, with a scanning rate of 0.002°/s and a step of 0.02°. These scanning parameters gave precise diffraction peaks of integral areas for calculating phase concentration. Before the XRD measurement step, the sintered ceramics were finely hand-ground. This step purposes to prevent any orientation of preferred grains within the material, and therefore to safeguard an arbitrary spatial distribution of the intensity of the reflection. 

### 2.2. Prediction of the Radiation Shielding Characteristics Using Monte Carlo Simulation 

The shielding properties of the fabricated ceramics were evaluated via two different methods: Theoretically, by means of the XCOM program and based on the NIST database created by Berger and Habull 1983, while the second method is Monte Carlo simulation (MC). The MC method, reported in many previous articles, proves its ability to predict the shielding behavior of any material currently using the ENDF/B-VI.8 database [25]. To obtain highly accurate results for the shielding parameters, an input file was created to accurately describe the experimental measurements. A 3D draw for the input file was exemplified in our previous work [26]. The mentioned 3D draw shows that an outer shielding cylinder of lead was employed to protect the geometry from the background activity. This outer cylinder has a thickness of 5 cm and a highest measurement of 35 cm. It is standing vertically along the Z direction. The outer cylinder contains various components such as radioactive source, collimators, a sample, and a detector. The radioactive source was set to emit γ photons in the interval of energy ranging between 0.015 and 15 MeV; it has a diameter of 1 cm and thickness of 0.3 cm. This radioactive source is surrounded by a collimator of lead with a highest point of 7 cm and a diameter of 5 cm. The collimator is used to absorb the scattered photons from the radioactive source and collimate the incident photons. The photons then act on the ceramic samples which have diameters of 2 cm and various thicknesses. In the distance between the ceramic sample and detector, another collimator is used to collimate the photons transmitted from the sample. The detector was assumed to be an F4 tally to record the number of photon flux per unit volume of the cell. The simulation ran out with NPS card 10^6^ historical. The output file recorded a relative error of ±0.1%. After that, all shielding parameters were calculated based on the mean track length of γ photons predicted by MC simulation, as discussed deeply in previous publications [26,27].

## 3. Results and Discussion

### 3.1. Phase Composition

**Figure 1:**
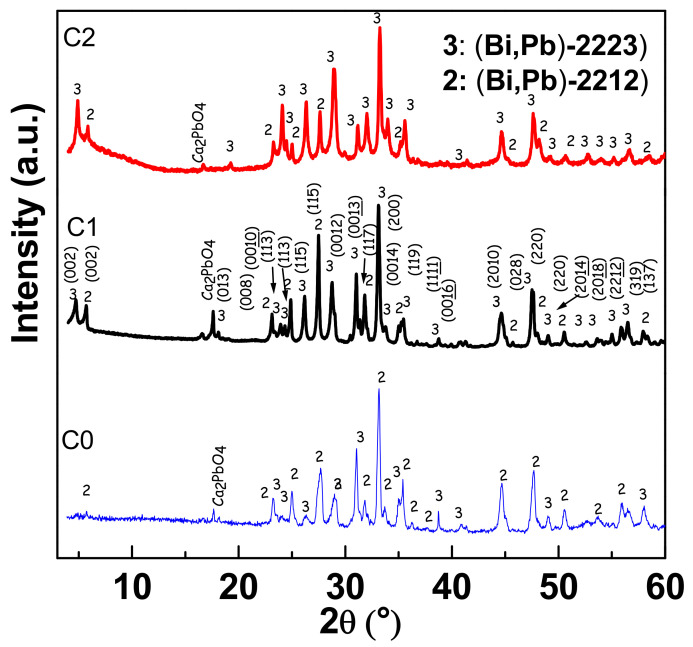
XRD patterns of C0 (blue spectrum), C1 (black spectrum), and C2 (red spectrum).

To identify the phase composition of our Bi (Pb)–Sr–Ca–Cu–O prepared ceramics and assess the development of phase transformation, X-ray measurements were taken in the 2*θ* range of 3–60°, and all the spectra are illustrated in Figure 1. Characteristic peaks matching the various phases were indicated on different peaks. In all spectra, the Bi-2223 and the Bi-2212 phases are presented by 3 and 2 abbreviations, respectively. To guess the volume fraction of each phase, the intensities of the XRD peak were considered, taking the relative intensities of the peaks in the pure compounds provided by the XRD file. The volume fraction for a given phase can be computed using the following equation:

(1) $$V\;\left(phase\;i\right)=\frac{\sum I\;phasei}{\sum I\;phasei+\sum I\;other\;phases}\times 100$$

where *phase i* is the Bi-2223 or Bi-2212 phase. In the first part of this work, we will study the influences of sintering and AgNO_3_ addition on the phase formation of Bi (Pb)–Sr–Ca–Cu–O ceramic.

#### 3.1.1. Effect of the Second Thermal Cycle

The blue XRD spectrum in Figure 1 corresponds to the C0 ceramic. This ceramic is the IC obtained after calcination and only one first step cycle. The analysis of these spectra indicates that this ceramic is predominantly composed of (Bi, Pb)-2212 phase (65.3 vol%), 34.7 vol% of (Bi, Pb)-2223 phase, and a trivial amount of Ca_2_PbO_4_ as the second phase. In the sintered ceramic C1, the second sintering step at 850 °C induces an increase in volume fraction of the (Bi, Pb)-2223 phase at the expense of the Bi-2212 phase. This is clearly distinguished by the typical (002) peak related to the (Bi, Pb)-2223 phase detected at 2*θ* = 4.72 in the black spectrum of Figure 1. This characteristic peak is not observed in the blue spectrum (C0 ceramic). This result seems to indicate that a large amount of the Bi-2212 phase converted into the (Bi, Pb)-2223 phase and that this transformation does not favor any new crystallographic orientation. The Bi-2212 phase is in the form of sheets obtained after the first sintering step, can function as a precursor, and changes chemically to convert to the (Bi,Pb)-2223 phase. Furthermore, this transformation can be explained by the conversion of the Bi-2212 phase in the occurrence of a liquid phase during the second sintering at 850 °C. This liquid phase is essential for the formation of the Bi-2223 phase. Yet, it should be appropriately transformed to Bi-2223 to increase the 2223 fraction [28]. Thus, the second sintering stage is favorable for converting liquid to the Bi-2223 phase. 

#### 3.1.2. Influence of AgNO_3_ Addition

**Figure 2:**
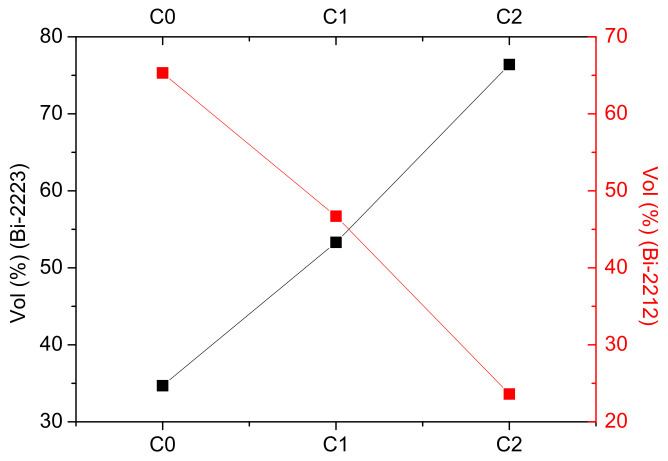
The volume fraction of Bi-2212 and Bi-2223 phases in different C0, C1, and C2 ceramics.

The red spectrum in Figure 1 corresponds to C2 ceramic obtained after two heat treatment cycles with AgNO_3_ addition. The analysis of this spectrum showed that the Bi-2223 is present as the main phase in this ceramic, while Bi-2212 appears as a minor phase. The concentration of the Bi-2223 phase is remarkably increased with AgNO_3_ addition. The volume fractions of Bi-2223 augmented from 53.3 vol% for C1 ceramic to 76.4 vol% for C2 ceramic (Figure 2). Meanwhile, Bi-2212 halved from 46.7 vol% for C1 ceramic to 23.6% for C2 ceramic. This sharp reduction in the volume fraction of Bi-2212 in the C1 sample indicates that the majority of this phase was converted to (Bi, Pb)-2223 phase. This phase conversion was fairly speedy with AgNO_3_ addition, compared to the ceramic prepared without AgNO_3_. The addition of AgnO_3_ significantly improves the (Bi, Pb)-2223 formation rate in ceramics. This signifies that the presence of AgNO_3_ led to a change in the homogeneity of the transient liquid composition, its viscosity, and the rate formation of the main phase. Hence, the addition of AgNO_3_ may most probably contribute to the creation of gas pockets which facilitate the liquid phase diffusion between the Bi-2212 sheets, thus promoting the proliferation of the nucleation sites and therefore the growth of the phase (Bi, Pb)-2223.

The crystallites size D of the different C0, C1, and C2 ceramics were determined using the Debby–Scherrer equation, as follows [29]:

(2) $$D=\frac{K\lambda }{\beta cos\theta }$$

where *K* is a dimensionless constant, β is the peaks’ width at half maximum intensity, and λ is the wavelength (λ=0.15406 nm).

**Figure 3:**
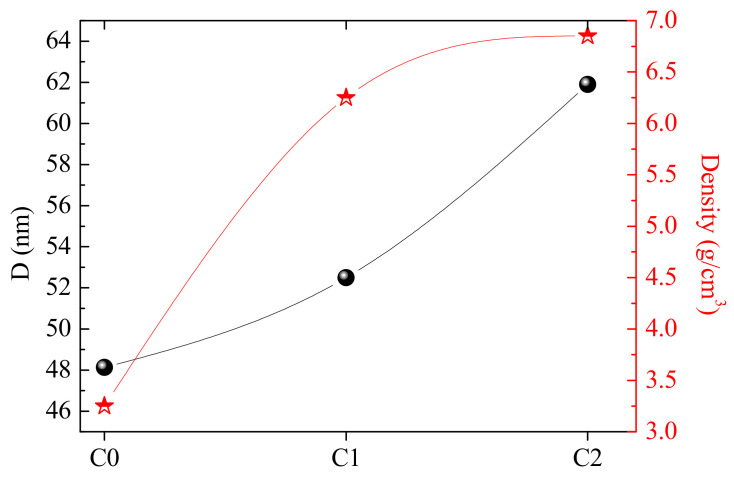
Crystallite size (D) and density (ρ) in C0, C1, and C2 ceramics. Round symbols correspond to D values and star symbols correspond to density values.

Figure 3 displays the evolution of the crystal size D_._ The crystal size shows a gradual increase from 48.15 nm for C0 to 52.50 for C1 ceramic, indicating the beneficial effect of the second sintering process in enhancing the bonding of BSCCO. When compared to the three as-prepared ceramics, C2 ceramics exhibits the highest value of crystallite size D (61.9 nm). This means that the addition of AgNO_3_ extremely strengthens the bonding between the BSCCO grains, positively affects the bond lengths, and increases the interplane coupling. It can then be assumed that the silver resides at the grain boundaries, filling the pores between the grains and further aiding in the conversion of the Bi-2212 phase to the Bi-2223 phase via the liquid phase.

The density ρ of the different ceramics was calculated. The different values are listed in Table 1, and their evolution is shown in Figure 3. It is clear that ρ has a similar variation as the crystallite size. The density ρ follows this order: C2 > C1 > C0. This implies that the sintering step increases the crystallite size and, consequently, leads to more dense ceramics. The highest value of ρ is obtained for C2 ceramic displaying the greatest crystallite size D. This can be due to the AgNO_3_, which may diffuse at grain boundaries, leading to improved interconnectivity and consequently resulting in the growth of grains and densification of the material. Our results are consistent with those reported by D. Sýkorová et al. [24]. Such a result is beneficial for the radiation shielding performances of the material. This will be deeply discussed in the next section of this work.

### 3.2. Radiation Shielding Parameters

**Figure 4:**
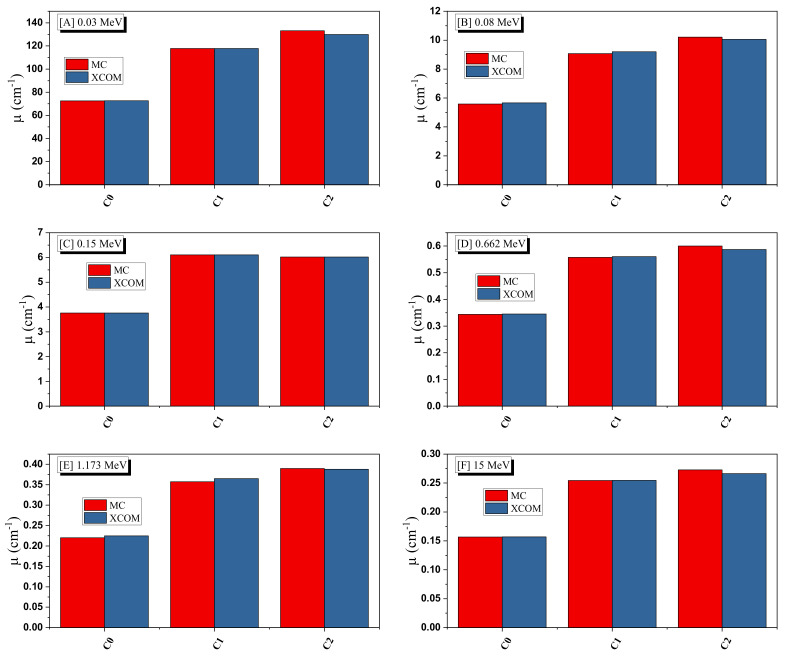
Comparison between the simulated and calculated linear attenuation coefficient for the fabricated ceramics at (**A**) 0.03 MeV, (**B**) 0.08 MeV, (**C**) 0.15 MeV, (**D**) 0.662 MeV, (**E**) 1.173 MeV and (**F**) 15 MeV.

To examine the radiation attenuation properties of the three prepared ceramics, the linear mass attenuation coefficients (µ) have been simulated by using the Monte Carlo simulation (MC). To validate the predicted µ obtained by MC, we used XCOM for the sake of comparison between the simulated and theoretically calculated µ. This is a very important step because we will use the MC results to predict the other crucial radiation attenuation parameters, such as the mean free path and others. The comparison will also allow for estimating of accuracy in the transmission geometry setup for MC. In Figure 4, we presented the comparison in the predicted µ values via MC and those determined by XCOM at some energies (0.03, 0.08, 0.15, 0.662, 1.173, and 15 MeV).

**Figure 5:**
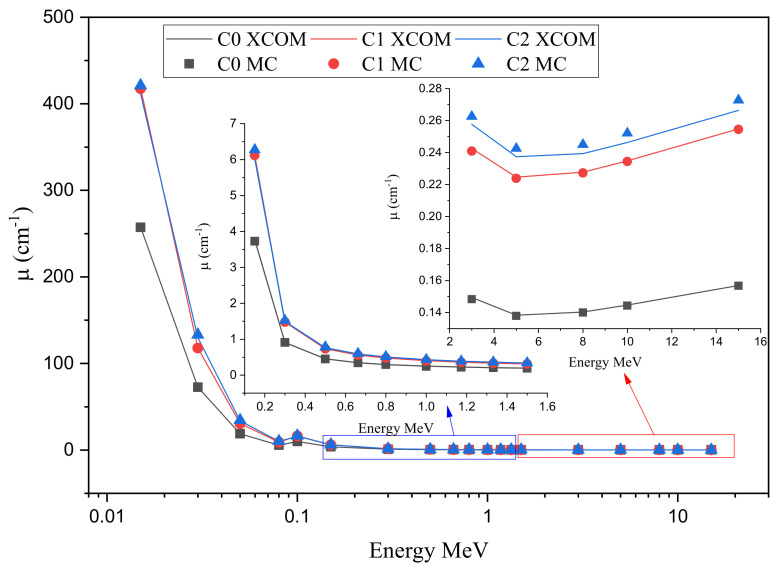
The linear attenuation coefficient of the fabricated ceramics samples.

For the three ceramics, good agreement is reported between the µ obtained in the two methods, and the difference in the µ between the MC and XCOM at each energy given in the figure is extremely small, and less than 3% in most cases. Accordingly, perfect compatibility is reported between the simulation and theoretical data. Hence, the present transmission geometry setup for MC was repeated for the same ceramics but at a wide energy range, and the results are given in Figure 5. The µ values are high at 15 keV (257.2 cm^−1^ for C0, 417.57 cm^−1^ for C1, and 421.16 cm^−1^ for C2). These values sharply dropped and are 72.58, 117.83, and 133.19 cm^−1^ at 30 keV, while at 50 keV, they are 18.80, 30.52, and 34.52 cm^−1^ for the same respective ceramics. Thus, the µ starts with high values and quickly declines with increasing energy. This is ascribed to the photoelectric effect. This effect is important for the low energy radiation and highly depends on the energy (inverse relation), so this explains the previous trend in the µ between 15 and 50 keV. This high reduction in the µ is not observed at higher energy, and the µ is changed very slowly with the energy between 0.3 and 1.5 MeV. This is due to the Compton scattering. For C0, the µ values are 0.452, 0.344, 0.294, and 0.244 cm^−1^ at 0.5, 0.662, 0.8, and 1 MeV. At 10 and 15 MeV, the µ has an opposite trend with the energy, i.e., we found a slight increase in the µ which is ascribed to the pair production process [30]. On the other hand, we can understand the influence of sintering and the effect of silver on the µ values. Let us compare the µ between the first and second samples (before and after sintering). We can see that the µ for the ceramic sample after sintering is much higher than the ceramics before sintering. Due to the high temperature and pressure during the sintering process, the grain boundaries were reduced. The density of the ceramics was notably enhanced, so we found that the µ for C1 is higher than that of C0. When we compare the µ for C1 and C2, we can see that the µ for C2 is higher than that of C1. This is because C2 contains AgNO_3_. Therefore, we can conclude that the AgNO_3_ improves the radiation attenuation performance for the prepared ceramics.

**Figure 6:**
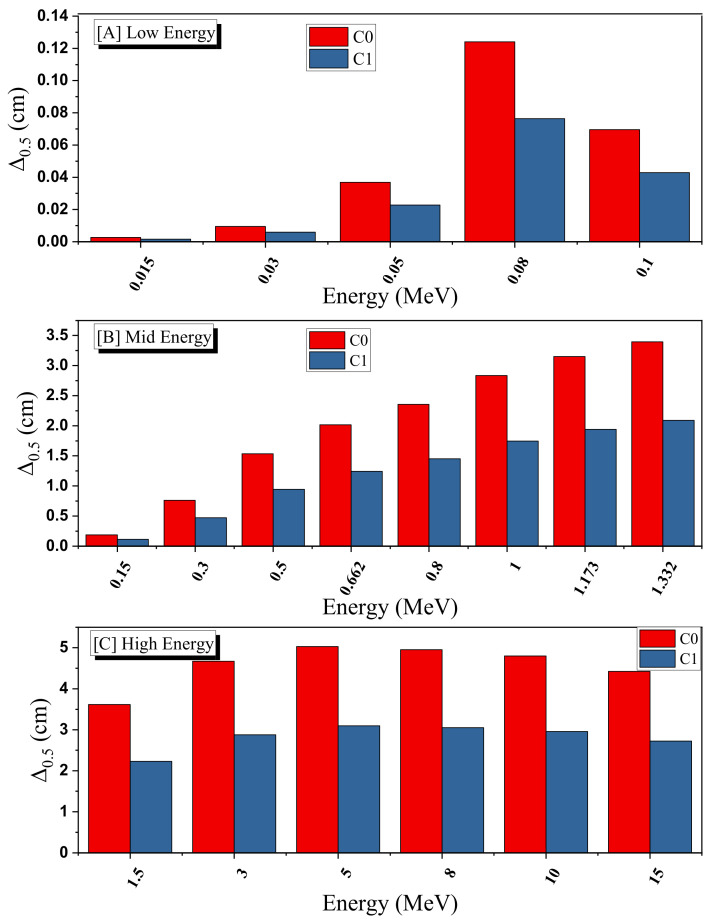
The effect of sintering on the half-value layer (Δ_0.5_, cm) at (**A**) low, (**B**) mid and (**C**) high energies.

**Figure 7:**
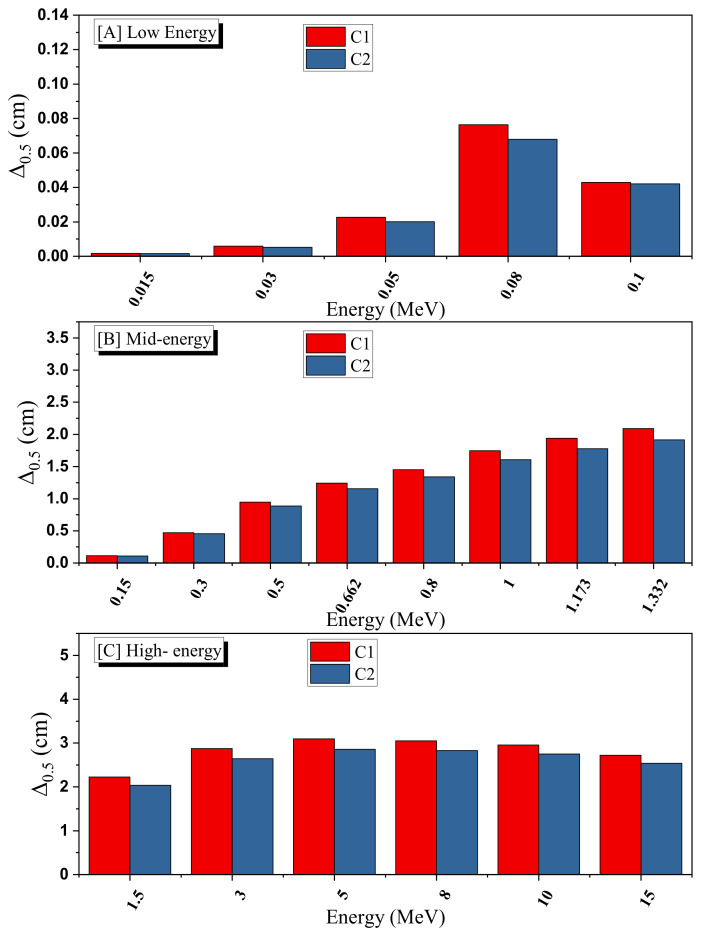
The effect of AgNO_3_ on the half-value layer (Δ_0.5_, cm) at various gamma-ray energies.

To the deep investigation of the influence of both the sintering and the effect of silver on the attenuation ability of the prepared ceramics, we plotted the half-value layer (Δ_0.5_) for C0 and C1 at low energies (between 0.015 and 0.1 MeV), moderate energy (0.15–1.332 MeV), and high energy (1.5–15 MeV) in Figure 6A–C. In addition, in Figure 7A–C, we plotted the (Δ_0.5_) for C1 and C2 at the same respective energies. Examining the data in Figure 6, we can see that, at the three selected regions, the Δ_0.5_ for C1 is smaller than that of C0. Therefore, it is reasonable to conclude that the sintering has a considerable influence on the thickness of the glass that is needed to attenuate the radiation. From Figure 6A, we see that the sintering has a weak effect on the Δ_0.5_ at 0.015 MeV, since this is very low energy, and thus, the ceramics without and with sintering can attenuate these low energy photons. Moreover, from the same subfigure, we can see that the Δ_0.5_ at 0.1 MeV has an opposite trend; the Δ_0.5_ is increased with increasing the energy, but at this energy, we found that the Δ_0.5_ is decreased (the Δ_0.5_ at 0.08 MeV is higher than that at 0.1 MeV). Both ceramics contain Bi and Pb, and it is known that at 0.1 MeV, the mass and linear attenuation coefficients of both elements are high (due to the K-absorption edge), and thus, the Δ_0.5_ is relatively small at this energy.

From Figure 7, it is obvious that the Δ_0.5_ for C2 is lower than that of C1. One must remember that the difference between both ceramics is the AgNO_3_. The reduction that occurred in the Δ_0.5_ for C2 should be mainly ascribed to enhancement in the density. As we note, the addition of AgNO_3_ in the C2 sample enhances the density, and this causes a reduction in the thickness of the sample that can attenuate the incoming radiation. However, the influence of the AgNO_3_ on the Δ_0.5_ is very small at low energy. At 0.015 MeV, the Δ_0.5_ for C1 and C2 are 0.00165 and 0.00164 cm, respectively (the difference is negligible). The difference in the Δ_0.5_ between C1 and C2 is notable at higher energies. Thus, we can conclude that C2 exhibits better attenuation performance than C0 and C1, and it is promising for radiation shielding applications.

**Figure 8:**
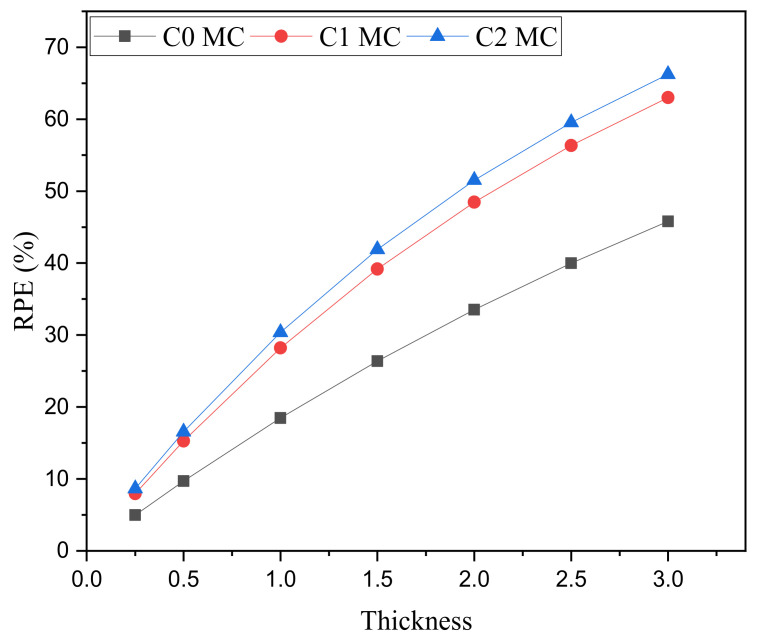
Variation in the radiation protecting efficiency of the fabricated ceramics versus the ceramics thickness at gamma ray energy of 1.332 MeV.

We investigated the influence of the thickness of the ceramics on the radiation shielding ability by evaluating the radiation protecting efficiency (RPE). This is obtained by applying the Lambert–Beer law and considering different thicknesses of each sample (i.e., 0.25, 0.5, 1, 1.5, 2, 2.5, and 3 cm). Figure 8 shows the variation in the RPE at the selected thicknesses for the three ceramics. The results are exhibited at one single energy (i.e., 1.332 MeV). With increasing thickness, the RPE ascends progressively from 4.97% for C0 with a thickness of 0.5 cm to 45.80% at 3 cm, from 7.95% to 63.0% for C1, and from 8.65 to 66.24% for C2. If we examine the RPE at fixed thickness, we see the following order: C2 > C1 > C0. This reaffirms our observation in the previous figures that the sintering has a positive influence on the attenuation ability of the prepared ceramics and causes an enhancement in the radiation shielding performance (since RPE for C1 is higher than the RPE for C0). The results also reaffirm that the addition of AgNO_3_ causes an improvement in the radiation shielding performance for these ceramics.

**Figure 9:**
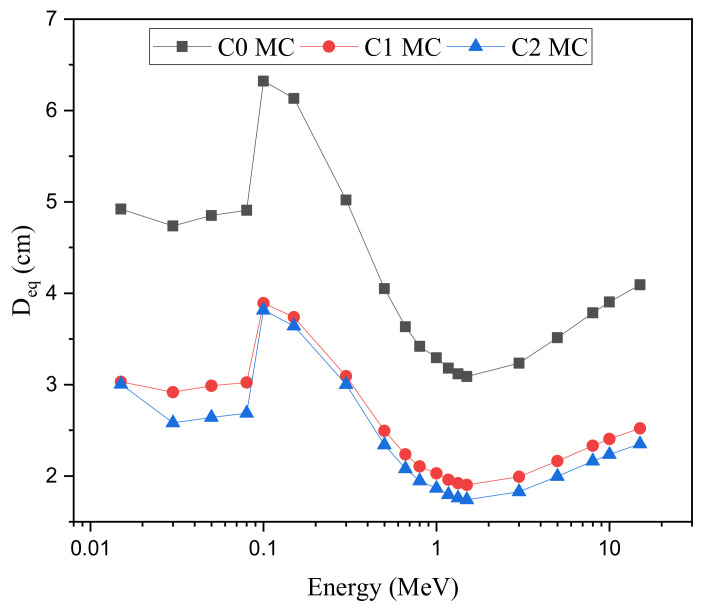
The fabricated ceramic’s equivalent thickness (D_eq_) variation versus the gamma-ray energy.

Based on the simulated µ values, the thickness of the fabricated ceramic equivalent to 1 cm of the pure lead element was calculated and plotted in Figure 9. At 0.015 MeV, the D_eq_ is 4.920, 3.031, and 3.005 cm for the ceramic samples C0, C1, and C2, respectively. The thicker D_eq_ thickness at the mentioned energy is received for the C0 sample with a density of 3.85 g/cm^3^ and without sintering. On the other hand, the thinner D_eq_ was achieved for both C1 and C2 with densities of 6.25 cm^2^/g and 6.85 cm^2^/g, respectively. The sintering process utilized in the preparation of samples C1 and C2 helps to increase the density of the fabricated ceramics due to the application of heat and pressure. This increase in the density of the fabricated ceramics has a positive effect on the photon’s passing resistance, so the µ values of the fabricated ceramics increase and D_eq_ decreases as a result. After that, the D_eq_ for all ceramics samples were slight decreases in the energy range between 0.015 and 0.08 MeV. A significant increase in the D_eq_ values was observed between 0.08 and 0.1 MeV, where the D_eq_ values reach to maximum and have values of 6.322, 3.893, and 3.816 cm from samples C0, C1, and C2, respectively. This high decrease is due to the X-ray K-absorption edges of lead, which appear at 0.088 MeV. At the mentioned energy, the µ value for pure lead has a high unnormal jump. Thus, the D_eq_ also has an unnormal jump to higher levels. After that, in the Compton scattering region, the D_eq_ regularly decreases with increasing the incident gamma photon energy. This behavior confirmed that the µ_Pb_ for lead decreases regularly compared to the sample’s µ values. Among the studied energy interval, the thinner D_eq_ obtained at 1.5 MeV takes values of 3.090, 1.903, 1.742 cm for samples C0, C1, and C2, respectively. This thickness confirmed that, at the mentioned energy, the shielding efficiency of the C1 and C2 is higher than 50% as compared to the pure lead element, while the ceramic sample C0 has a shielding efficiency of about 33% of the pure lead. This result clearly shows the effect of sintering on the shielding capacity of the fabricated ceramics. For E > 1.5 MeV, the D_eq_ began to increase slightly with energy, which confirmed that at very high energy, the lead µ values increase slightly compared to the fabricated ceramic’s µ values.

## 4. Conclusions

We have conducted a comparative investigation of the formation phase and radiation protecting properties of three (Bi, Pb)-2223 ceramics. C0 ceramic was obtained after calcination and one heat cycle. C1 and C2 ceramics were obtained after two cycles of sintering; AgNO_3_ was added at the beginning of the final sintering step to form a C2 ceramic. We showed growth of (Bi, Pb)-2223 phase during the second thermal cycle, while the Bi-2212 volume fraction decreased successively. Moreover, the addition of AgNO_3_ was significantly effective in further increasing the Bi-2223 phase from 53.3 vol% for C1 ceramic to 76.4 vol% for C2 ceramic and increasing the amount of grain interfacial conduction. The obtained results indicated that the sintering conditions and the addition of AgNO_3_ are among the most important determinants of the degradation rate of (Bi, Pb)-2212 and the formation of the liquid phase and, thus, Bi-2223′s transformation. The radiation shielding parameters of the different ceramics were calculated using Monte Carlo simulation. The difference between the MC and XCOM methods is extremely small, and less than 3% in most cases. We studied the influence of the sintering on the µ, Δ_0.5,_ and other shielding factors by comparing C0 with C1, while we studied the influence of AgNO_3_ on the shielding factors by comparing C1 with C2. The comparison between the µ for C0 and C1 proved that the sintering has a positive effect on the radiation shielding competence for the prepared ceramics. When we compared µ for C1 and C2, we found that the AgNO_3_ improves the radiation attenuation performance for the prepared ceramics. On the other hand, with increasing the thickness, the RPE ascends progressively from 4.97% for C0 with a thickness of 0.5 cm to 45.80% at 3 cm, from 7.95% to 63.0% for C1, and from 8.65 to 66.24% for C2. When we examined the RPE at fixed thickness, the following order was reported: C2 > C1 > C2, which reaffirms that the sintering and AgNO_3_ addition have a positive influence on the attenuation ability of the prepared ceramics and causes an enhancement in the radiation shielding performance. The results obtained are very motivating for the prospect of using the Bi (Pb)–Sr–Ca–Cu–O ceramic with the addition of AgNO_3_, using two thermal cycles, for radiation protection applications.

## Figures and Tables

**Table 1:** Sintering conditions of C0, C1, and C2 ceramics.

Ceramic Code	Sintering Conditions				Density (g/cm^3^)
	Calcination (930 °C, 24 h)	First Sintering Step (835 °C, 140 h)	Second Sintering Step (850 °C, 156 h)	AgNO_3_ Addition	
C0	×	×	-	-	3.25
C1	×	×	×	-	6.25
C2	×	×	×	×	6.85

## Data Availability

All of the relevant data are within this paper.
